# An Unusual Manifestation of Blastic Plasmacytoid Dendritic Cell Neoplasm as a Testicular Tumor

**DOI:** 10.1155/2019/9196167

**Published:** 2019-10-07

**Authors:** Daniel M. Pak, Maria S. Tretiakova

**Affiliations:** University of Washington School of Medicine, 1959 NE Pacific Street, Seattle, WA 98195, USA

## Abstract

Blastic plasmacytoid dendritic cell neoplasm (BPDCN) is a clinically aggressive hematologic malignancy arising from precursors of plasmacytoid dendritic cells that represent less than 1% of hematological malignancies. BPDCN initially presents with cutaneous involvement and a characteristic immunophenotype of CD4, CD56, and CD123 co-expression. Upon disease progression, BPDCN shows a strong predilection for bone marrow, peripheral blood, and lymph nodes, whereas manifestations in visceral organs are rare. Significant heterogeneity in clinical presentation and immunophenotypic profile makes BPDCN challenging to diagnose without an integrated approach based on patient history, clinical features, tumor pathology, and comprehensive immunohistochemical studies. Herein we report the first case of relapsed BPDCN manifesting as a unilateral testicular tumor.

## 1. Introduction

The 2008 World Health Organization (WHO) classification of tumors of hematopoietic and lymphoid tissues recognized blastic plasmacytoid dendritic cell neoplasm (BPDCN) as a distinct entity characterized by the malignant proliferation of precursors of plasmacytoid dendritic cells [1]. BPDCN can present in all age groups, but is more common in adults with a median age of presentation above 60 years and has a slight male predominance with a male to female ratio of 2.5 : 1 [2]. Initial presentation most commonly involves skin lesions with or without bone marrow involvement and leukemic dissemination, but cases with fulminant leukemia without cutaneous manifestation have been described [3, 4]. Prognosis is poor with most cases rapidly and uniformly fatal [4]. Initial manifestations of BPDCN without cutaneous involvement are extremely rare with a few case reports described in the literature. We report a case of a 54-year-old man who presented with a unilateral testicular mass mimicking a primary testicular neoplasm, ultimately diagnosed as relapsed BPDCN.

## 2. Case Presentation

The patient was a 54-year-old man who presented with nontender, left-sided scrotal swelling. Scrotal ultrasound showed a hypoechoic, hypervascular, left intrastesticular mass with microcalcifications (3.2 × 2.4 × 3.2 cm) concerning for a primary testicular neoplasm. For definitive diagnosis, the patient underwent a left radical inguinal orchiectomy.

**Figure 1:**
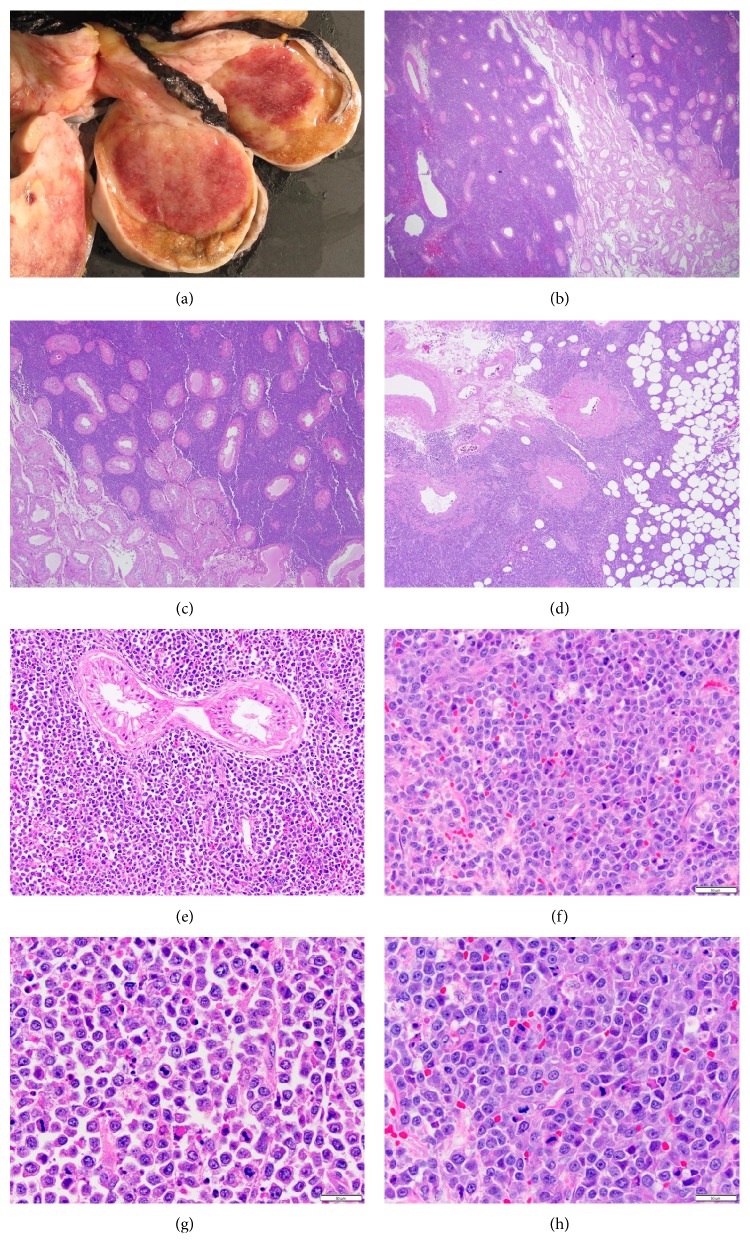
Gross and representative H&E images. Gross findings showed a tan-red, hemorrhagic, well-circumscribed circular mass with focal areas of tan-white friable necrosis (a). At low power, diffuse, solid sheets of densely packed neoplastic cells infiltrating the testicular parenchyma, hilar soft tissue, epididymis, and spermatic cord (b–d) with sparing of the seminiferous tubules (e) were seen. At high power (f–h), medium-sized neoplastic cells showed blastoid morphology, scant agranular cytoplasm, irregular nuclei with fine to vesicular chromatin, and small nucleoli. Increased mitotic activity with atypical mitotic figures, areas of necrosis, and abundant apoptotic debris were seen.

On gross examination, the testicular mass was serially sectioned to reveal a tan-red, hemorrhagic, well-circumscribed circular mass (5.0 × 4.0 × 3.4 cm) with focal areas of tan-white friable necrosis (Figure 1(a)). The mass spared the epididymis (2 × 1.2 × 3 cm) and abutted the tunica albuginea. The remaining uninvolved testis parenchyma was tan-yellow and unremarkable.

Histologically, the testicular mass showed diffuse, solid sheets of densely packed neoplastic cells infiltrating the testicular parenchyma, hilar soft tissue, epididymis, and spermatic cord (Figures 1(b)–1(d)) with sparing of the seminiferous tubules (Figure 1(e)). The tumor consisted of medium-sized neoplastic cells with blastoid morphology, scant agranular cytoplasm, irregular nuclei with fine to vesicular chromatin, and small nucleoli. The tumor exhibited increased mitotic activity with atypical mitotic figures, areas of necrosis, and abundant apoptotic debris (Figures 1(f)–1(h)). Based on morphology and patient age, the neoplastic cells seemed most consistent with either a lymphoma or spermatocytic tumor with anaplastic features.

**Figure 2:**
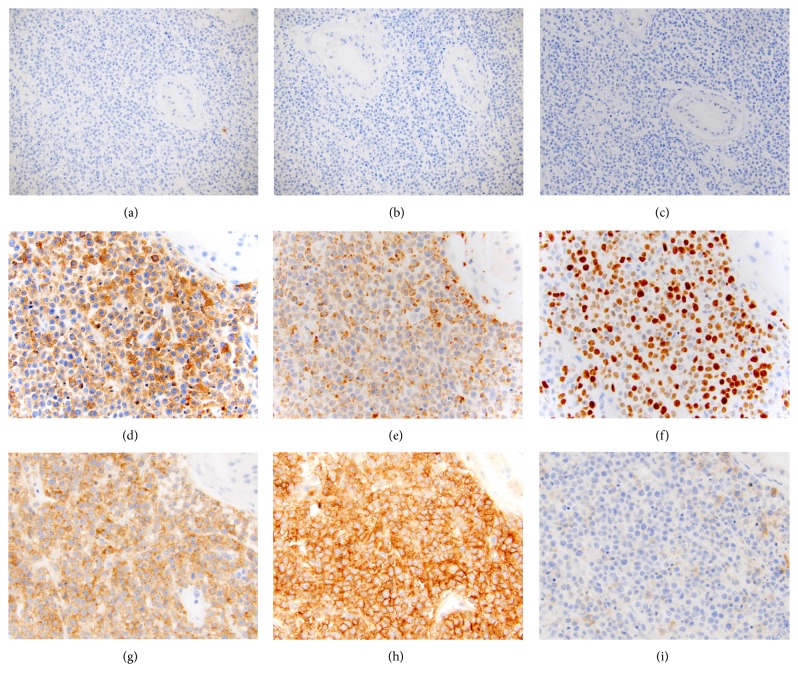
Representative immunohistochemistry images. Initial IHC panel of CD3 (a), CD20 (b), and SALL4 (c), was negative and argued against a diagnosis of lymphoma, primary germ cell tumor, or epithelial neoplasm. The neoplastic cells were weakly positive for CD45 (d), and granularly positive for CD68 (e), with dot-like pattern. Ki-67 (f), was expressed in 80% of cells. The diagnosis of BPDCN was consistent with positive CD4 (g), CD56 (h), and weak CD123 (i).

A preliminary immunohistochemistry (IHC) panel of CD3, CD20, AE1/AE3, and SALL4 was performed (Figures 2(a)–2(c)). The neoplastic cells were negative for all four markers, arguing against a diagnosis of lymphoma, primary germ cell tumor, or epithelial neoplasm. To further characterize the neoplasm, a second IHC panel of CKIT, CD45, CD68, S100, Ki-67, and CD138 was performed (Figures 2(d)–2(f)). The neoplastic cells were weakly positive for CD45 and positive for CD68 with granular dot-like pattern. Ki-67 was expressed in 80% of cells. CKIT, S100, and CD138 were negative. The positive CD45 and CD68 were suggestive of a hematopoietic neoplasm with histiocytic differentiation.

At this time, the patient's chart was reviewed, which showed a history of BPDCN that initially presented as scalp nodules. The patient had received chemotherapy and a bone marrow transplant less than three months ago. Subsequently, a third IHC panel of CD4, CD56, and CD123 was performed (Figures 2(g)–2(i)). The neoplastic cells were diffusely positive for CD4 and CD56, and CD123 was positive in only rare cells, supporting the diagnosis of BPDCN.

After the diagnosis of relapsed BPDCN, the patient was treated with five rounds of pralatrexate with palliative intention. Disease progression was evidenced by the presence of diffuse joint pain and inguinal lymphadenopathy. The patient expired three months later.

## 3. Discussion

BPDCN is a diagnosis of exclusion, and substantial heterogeneity in clinical presentation can make the diagnosis very challenging. In the natural history of BPDCN, the skin is typically the first affected site, where it usually remains confined until a rapid second step involving leukemic spread and multiorgan involvement, eventually leading to death [5]. Involvement of the tonsils, liver, soft tissues, paranasal cavities, lungs, eyes, and central nervous system has been described [2]. However, initial manifestations of BPDCN without cutaneous involvement are extremely rare. Dhariwal et al. recently described a case of a 13-year-old male who initially presented with BPDCN manifesting as a testicular mass without any cutaneous manifestation followed by leukemia-like symptoms with bone marrow involvement [6]. Lee et al. described two cases of adolescent females who initially presented with BPDCN manifesting as nasal cavity masses with leukemic involvement without any cutaneous manifestation [7].

In addition to variable clinical presentation, significant heterogeneity in immunophenotypic profile exists. Given this variability, no consensus has been established on the minimum IHC criteria. BPDCN is typically characterized by positive CD4, CD56, and CD123 [8]. However, several other lineage-specific antigens may be frequently expressed. For example, CD68, an antigen expressed by granulocytes and histiocytes, is present in about one half of cases [3]. In addition, typical markers of BPDCN, such as CD4 and CD56, may be absent. The link between clinical heterogeneity and immunophenotypic expression of BPDCN tumor cells has not yet been established [3].

Furthermore, because of the overlap of the immunophenotypic characteristics with other hematopoietic malignancies, extensive IHC analyses are often necessary. The differential diagnoses to consider include myeloid sarcoma/acute myeloid leukemia (AML), T cell lymphoblastic lymphoma/leukemia, NK cell lymphoma/leukemia, and some mature T cell lymphoma/leukemia [3, 9].

A possible mechanism by which BPDCN could manifest as a testicular mass may be comparable to initial presentations of myeloproliferative disorders, myelodysplastic disorders, and AML as extramedullary myeloid cell tumors (EMCT). Indeed, in the 2008 WHO classification, BPDCN is quoted after AML, reflecting that the gene signature of the precursor blastic plasmacytoid dendritic cells is much closer to that of myeloid than lymphoid precursors [5]. EMCT are solid tumor masses consisting of immature or mature myeloid cells and can involve any organ and occur at any age [10]. McIlwain et al. described a case of a 21-year-old male with AML initially manifesting as a testicular mass [10]. Interestingly, testicular relapse in adults with an established diagnosis of AML has been reported with an incidence between 1% and 2% of cases [11].

Further drawing similarities to BPDCN, the most commonly affected site for EMCT is the skin. This skin tropism may be related to the expression by both BPDCN and AML cells of skin-migration markers such as CD56 and CLA [5, 12]. In addition, several studies have indicated that expression of CD56 and CD4 in AML cells increases the propensity for the formation of EMCT [10]. Thus, the expression of CD56 and CD4 by BPDCN cells may also contribute to a similarly increased propensity for the formation of extracutaneous solid tumors.

In conclusion, we present the first case of relapsed BPDCN presenting as a unilateral testicular tumor in an adult patient. Nonspecific morphology, overlapping immunophenotypic profile, and rarity of this tumor make BPDCN an especially challenging diagnosis. The pathologist must use an integrated approach based on patient history, clinico-pathologic findings, and extensive IHC analyses for accurate diagnosis.
